# Triplex Real-Time PCR Approach for the Detection of Crucial Fungal Berry Pathogens—*Botrytis* spp., *Colletotrichum* spp. and *Verticillium* spp.

**DOI:** 10.3390/ijms21228469

**Published:** 2020-11-11

**Authors:** Dominika G. Malarczyk, Jacek Panek, Magdalena Frąc

**Affiliations:** Institute of Agrophysics, Polish Academy of Sciences, Doświadczalna 4, 20-290 Lublin, Poland; d.malarczyk@ipan.lublin.pl (D.G.M.); j.panek@ipan.lublin.pl (J.P.)

**Keywords:** D2LSU, *Botrytis* spp., *Colletotrichum* spp., *Verticillium* spp., real-time PCR, strawberry pathogens, rapid detection

## Abstract

Phytopathogens cause undeniably serious damage in agriculture by harming fruit cultivations and lowering harvest yields, which as a consequence substantially reduces food production efficiency. Fungi of the *Botrytis*, *Colletotrichum* and *Verticillium* genera are a main concern in berry production. However, no rapid detection method for detecting all of these pathogens simultaneously has been developed to date. Therefore, in this study, a multiplex real-time PCR assay for this purpose was established. Universal fungal primers for the D2 region of the large subunit ribosomal DNA and three multiplexable fluorogenic probes specific for the chosen fungi were designed and deployed. The triplex approach for the molecular detection of these fungi, which was developed in this study, allows for the rapid and effective detection of crucial berry pathogens, which contributes to a more rapid implementation of protective measures in plantations and a significant reduction in losses caused by fungal diseases.

## 1. Introduction

Fungal pathogens cause severe destruction in the food industry. Phytopathogenic diseases induce economic losses by reducing yield and also by decreasing fruit survival rates in cold storage. Some exceedingly harmful fungi which cause food spoilage not only in Europe, but also in Australia and America are species belonging to the *Botrytis*, *Colletotrichum* and *Verticillium* genera [1,2,3,4,5,6,7]. In recent years an increase of both the demand and production of organic fruit has been observed. The increase in fruit production is connected with the problem of lowering quality of crops, caused by the occurrence of fungal diseases in soft fruits plantations [5,6]. 

The early detection of soil-borne diseases is very important for both economic reasons and nature protection. However, traditional methods of fungal identification are time consuming and often inaccurate [8]. Additionally, they require trained and experienced staff for precise identification, as fungal pathogens often attack a wide range of plants; moreover, they frequently have an ambiguous appearance on their hosts. In contrast, molecular techniques allow for the detection of crucial pathogens with a high degree of precision, without specific molecular knowledge, and even before the manifestation of disease [8]. Moreover, only quick and accurate identification of the pathogen guarantees the timely implementation of suitable plants protection against pathogenic agent and reduction of losses for farmers or other manufacturers and fruit processing companies [5,6].

The molecular technique most frequently used for the identification of microorganisms is the Polymerase Chain Reaction (PCR). However, the final results of the analysis are not obtained immediately after the reaction, as the method requires further steps, such as an electrophoresis and the sequencing of the obtained amplicons, in order to interpret the results. Another disadvantage of the reaction is that the assay requires specific thermal conditions provided by thermocyclers [9].

For those reasons, various modifications of the method, designed to omit the inconvenient steps mentioned above, have already been developed. Quantitative PCR (qPCR) allows for the quantification of the amplified genetic material in real time [9,10,11]. The exclusion of many laboratory steps, used to obtain the final results of the analysis, considerably shortens the time for detection. This allows for a more rapid implementation of the relevant protective measures in the plantation. The hybridization of multiple fluorogenic probes with TaqMan chemistry [12] in the same reaction tube allows for the simultaneous detection of multiple targets in one reaction [13], which additionally decreases the time required in the laboratory for the acquisition of final results.

Isothermal methods of PCR, such as Loop-mediated isothermal amplification (LAMP) [14], Polymerase spiral reaction (PSR) [15], Rolling circle amplification (RCA) [16] and Cross-priming amplification (CPA) [17], have likewise been used more frequently in recent years. These methods are simpler and cheaper to deploy, as the use of a water bath or thermoblock is sufficient to carry out the reaction. Nevertheless, they require a larger number of specific enzymes and a greater amount of primers; moreover, they are troublesome to develop and multiplex.

Fragments of nuclear ribosomal DNA (rDNA) are commonly used for molecular identification and phylogenic purposes for different fungal microorganisms [18]. Primers covering Internal Transcribed Spacer (ITS) and Intergenic Spacer (IGS) regions are for the most part routinely taken into consideration [7,19,20,21]. These markers are commonly applied in qPCR assays [13,22,23,24,25,26]. Likewise, the LSU rDNA D1-D2 sequences have been used in the purpose of identification of fungal species many times [8,27,28,29,30].

The aim of this study was to develop the real-time PCR method for the detection of *Botrytis* spp., *Colletotrichum* spp. and *Verticillium* spp. using newly designed universal primers which amplify different fragments of rDNA—the D2 region of the large subunit ribosomal DNA. Additionally, we designed three multiplexable TaqMan-type hydrolysis probes, specific for the chosen fungi within this region, and tested the developed method on environmental samples.

## 2. Results

### 2.1. Phytopathogenic Microorganisms

**Figure 1:**
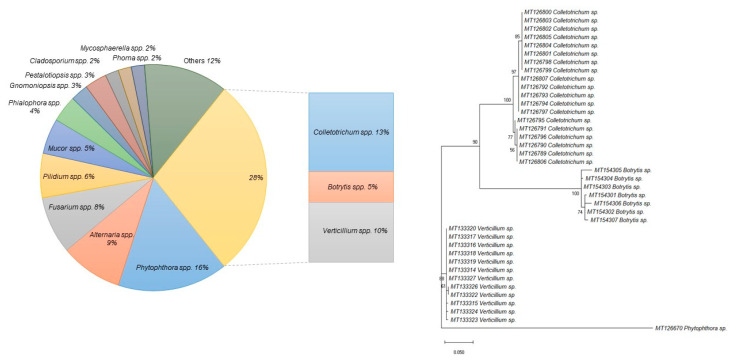
A pie chart representing the percentage of different fungi in the whole group of microorganisms isolated from organic plantations of strawberry in the course of this study. The evolutionary history of the four phytopathogens chosen for the development of the multiplex real-time PCR was inferred by using the Maximum Likelihood method based on the Tamura-Nei model [31]. The tree with the highest log likelihood (–1545.8023) is shown. The percentage of trees in which the associated taxa are clustered together are shown next to the branches. Initial tree(s) for the heuristic search were obtained automatically by applying the Maximum Parsimony method. The tree is drawn to scale, with branch lengths measured in the number of substitutions per site. The analysis involved 39 nucleotide sequences. There was a total of 527 positions in the final dataset. Evolutionary analyses were conducted in MEGA7 [32].

A wide range of fungal pathogen isolates, derived from organic strawberry plantations located in Poland, were included in this study (Figure 1, Supplementary Table S1). Within this group of isolates, 28% of them (7 *Botrytis* spp., 19 *Colletotrichum* spp., 12 *Verticillium* spp.) were fungi selected for the design of primers and fluorogenic probes for novel multiplex real-time PCR. After that, four isolates, one representative of each genus, *Botrytis*, *Colletotrichum*, *Verticillium*, and one strain of *Phytophthora* sp., as a control organism that should not be detected, were selected for the development of the multiplex real-time PCR detection approach, in order to evaluate the specificity and the detection limit of the real-time PCR assays for target pathogens. The non-fungal *Phytophthora* sp. pathogen was selected as a control group due to the fact that those four microorganisms (*Botrytis* spp., *Colletotrichum* spp., *Verticillium* spp. and *Phytophthora* spp.) often cooccur in the fruit plantations, as in our experimental fields we retrieved strains from. The neighbor-joining tree of the 39 tested strains, based on the phylogenetic analysis of DNA sequences of the D2 LSU region, have indicated that the strains belonging to the genus *Colletotrichum* were clustered together in a monophyletic clade, supported by high bootstrap values. Similarly, the *Botrytis* spp. strains clustered together, and *Verticillium* spp. isolates were grouped in a separate cluster.

### 2.2. Specificity of the Fluorogenic Probes

**Figure 2:**
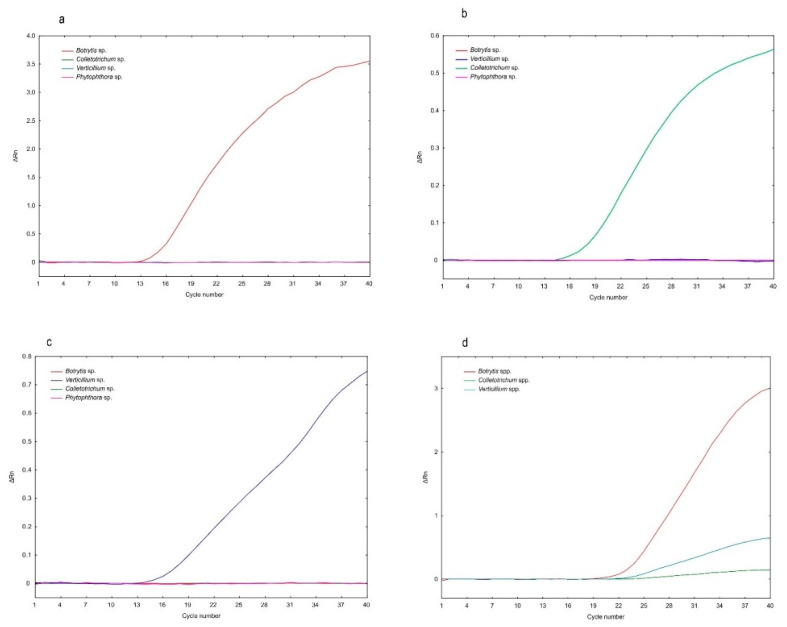
Amplification plots of designed singleplex and multiplex real-time PCR. (**a**–**c**): Specificity of the fluorogenic probes in singleplex reactions, carried out on DNA isolated from pure strain cultures. The amplification plots were created as mean values of ΔRn after each cycle of three biological repeats for the reaction (*n* = 3). (**d**) Multiplex reaction carried out with the DNA extracted from an environmental sample 245/19 (strawberry fruit).

Each of the three of the designed fluorogenic probes were only specific to the organism it was designed for and did not hybridize with the DNA of the remaining fungi (Figure 2a–c). The use of designed oligonucleotide primers and molecular probes to carry out the polymerase chain reaction with real-time detection under strictly defined conditions enabled the amplification of the product and the detection of *Botrytis* spp., *Colletotrichum* spp. and *Verticillium* spp. fungi, respectively. The specific detection of the obtained amplification products was carried out by noting fluorescence at 520 nm (Filter 1 of 7500 Fast system), 576 nm (Filter 2) and 667 nm (Filter 3), after excitation with light at 495 nm, 544 nm and 643 nm, for the abovementioned fungal plant pathogens, respectively (Table 1).

In singleplex real-time PCR (Figure 2a–c), the amplification curve analysis revealed the primer pair with each specific molecular probe (Table 1) and amplified a single product for its target genus and no product for the other three fungal genus, thereby indicating the specificity of the molecular probes to detect their corresponding to the *Botrytis*, *Colletotrichum* and *Verticillium* genus.

When the primer pair and the three probes were mixed together for multiplex real-time PCR, the amplification curve of each tested fungal genus (*Botrytis*, *Colletotrichum* and *Verticillium*) was visible, thereby indicating that all three fungal pathogens were detectable in one reaction (Figure 2d).

**Figure 3:**
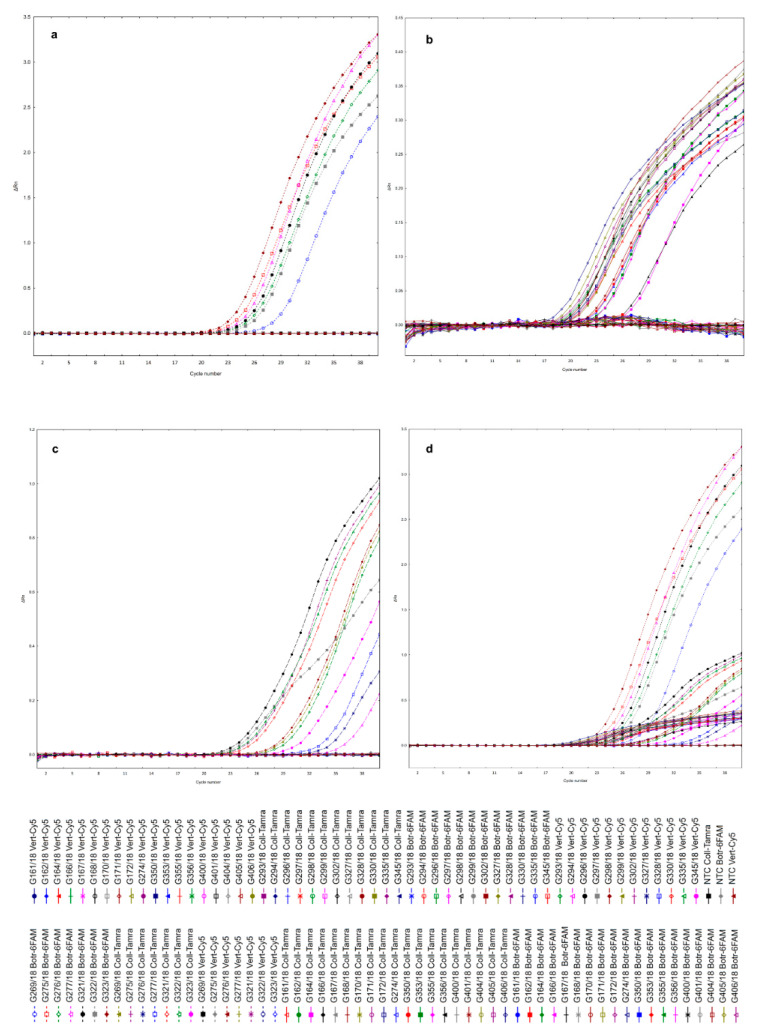
Amplification plots of designed triplex real-time PCR. Specificity of the fluorogenic probes (Botr-6FAM, Coll-Tamra, Vert-Cy5) in triplex reactions, carried out on DNA isolated from pure strain cultures of: (**a**) *Botrytis* spp. (7 isolates); (**b**) *Colletotrichum* spp. (19 isolates); (**c**) *Verticillium* spp. (12 isolates); (**d**) All tested isolates of *Botrytis* spp., *Colletotrichum* spp., *Verticillium* spp. (38 isolates). Explanations: NTC—non-template control.

Neither false positive nor false negative reactions were observed in the real-time PCR assays. The triplex real-time PCR assay with developed fungal pathogens specific probes for *Botrytis* spp., *Colletotrichum* spp. and *Verticillium* spp. detected all 7, 19 and 12 fungal isolates, respectively (Figure 3). The assay also did not cross-react with DNA from the other pathogens, including non-specific organisms. It means that the developed probe labeled with 6FAM dye for detection of *Botrytis* spp. detected only these pathogens, but DNA of *Colletotrichum* spp. and *Verticillium* spp. was not amplified with using this specific probe. High specificity of the fluorogenic probes labeled with Tamra and Cy5 for the detection of *Colletotrichum* spp. and *Verticillium* spp., respectively, was also noted. Likewise, the real-time PCR assay for the tested pathogens produced negative results for non-template control (NTC) without DNA. The results indicated that the fluorogenic probes developed in this study appeared to be specific to the three target fungal pathogens (Figure 3), and moreover, according to in silico analysis, appeared to not be specific to the diverse fungal and oomycete representatives (Table S2).

### 2.3. Optimization of Multiplex Real-Time PCR

In order to optimize multiplex real-time PCR and evaluate whether the sensitivity of the detection was associated with the concentrations of probes and primers for selected fungal strawberry pathogens, the mean Cq values for each concentration of each probes and primers were determined and the results are presented in Table 2.

The first stage of this study included the optimization of the fluorogenic probes concentration with standard concentration of primers (0.4 µM). The results indicated the most stable positive signal of amplification for 0.15 µM of fluorogenic probes concentration, exhibiting the following values of Cq ± SD cycles 14.084 ± 0.377, 19.286 ± 0.152 and 18.235 ± 0.417 for *Botrytis* spp., *Colletotrichum* spp. and *Verticillium* spp., respectively (Table 2). Even though higher probe concentrations often showed lower Cq values, in most cases the variation in the Cq signal for all of the tested fungal pathogens was excessive with a maximal SD of 0.895, 0.733 and 5.697 cycles for *Botrytis* spp., *Colletotrichum* spp. and *Verticillium* spp., respectively (Table 2). 

In the following step, various concentrations of primers were tested with probes concentration (0.15 μM) selected in the first phase of the experiment. The lowest primers concentration (0.1 μM) was less sensitive across all fungal pathogens, compared with the higher concentrations of primers, and it was also associated with a higher degree of variability (Table 2, Supplementary Figure S1). This primers’ concentration yielded the highest Cq values along with an unacceptably SD value (21.421 ± 4.103) for *Botrytis* spp.; moreover, for the other two fungi (*Colletotrichum* spp. and *Verticillium* spp.), the Cq values were not calculated and detection was not possible. Higher concentrations of primers were more sensitive, yielding lower Cq values for all tested fungal pathogens. However, different primer concentrations exhibited different degrees of variation in SD for triplicate measurements across the various fungal pathogens. Across all tested primers concentrations, 0.3 µM yielded a mean Cq ± SD (*n* = 3) of 16.478 ± 0.172, 18.777 ± 0.142 and 16.565 ± 0.146 for *Botrytis* spp., *Colletotrichum* spp. and *Verticillium* spp., respectively. These results were the least variable. For the other higher primer concentrations, even if the Cq values were lower, the SD values increased drastically, especially for *Verticillium* spp., indicating a lower reproducibility of amplification.

Within the tested combinations of primers and fluorogenic probes concentrations, the most stable amplifications results were provided by the 0.15 μM probes and the 0.3 μM primers mixture for all of the tested fungal pathogens (Table 2). 

### 2.4. Detection Limit of Multiplex Reaction

The evaluation was performed by diluting the DNA samples of the *Botrytis* spp., *Colletotrichum* spp. and *Verticillium* spp. isolated from pure strain cultures. Serial 2-fold dilutions of 5 pg/µL to 39 fg/µL of the DNA of each of the target strawberry pathogens were used to test the sensitivity of the multiplex real-time PCR assays. The detection limit was defined as the lowest amount of the targeted DNA that it was possible to detect with the method. The Cq values for 5 pg/µL DNA from each of the three target pathogens were 28.222 (SD ± 1.659), 31.144 (SD ± 1.106) and 30.659 (SD ± 0.902) for *Botrytis* spp., *Colletotrichum* spp. and *Verticillium* spp., respectively. The results indicated that the Cq values increased for higher DNA dilutions of the tested samples, but SD decreased, indicating a higher stability of strawberry pathogens detection (Table 3). Positive results with the lowest DNA dilution (39 fg/µL) for the *Botrytis* spp., *Colletotrichum* spp. and *Verticillium* spp. were visible at the amplification plots. However, Cq values were calculated for 39 fg/µL template DNA from *Botrytis* spp. (33.276 ± 0.286) and *Verticillium* spp. (36.267 ± 0.610), but for *Colletotrichum* spp. Cq values (36.892 ± 0.731) were obtained for 156 fg/µL template DNA, and for lower DNA amounts, Cq values were not obtained. Therefore, the detection threshold for two of the pathogens was set at 39 fg/µL for *Botrytis* spp. and *Verticillium* spp., and at 156 fg/µL for *Colletotrichum* spp. (Table 3).

### 2.5. Detection of Fungal Pathogens in Artificially Infested Environmental Samples

In the conditions of our study, the FastDNA Spin Kit for Feces (MP Biomedicals, Solon, OH, USA) was the most effective DNA isolation method for the detection of fungal strawberry pathogens from environmental samples. The real-time PCR assay detected *Botrytis* spp. and *Verticillium* spp. in all eight infected soil samples. The presence of *Colletotrichum* spp. was confirmed in two infested soil samples, when contamination was at the level of 5000 and 10,000 spores after 24- and 48-incubation hours, respectively (Table 4). There was no amplification of *Colletotrichum* spp. DNA from the soil samples inoculated with 1000 and 500 spores per 1 g. The Cq values ranged from 22.491 to 28.166 for the detection of *Verticillium* spp., and *Botrytis* spp. in artificially infested soil after 24-incubation hours, respectively. However, the Cq values for the detection of *Colletotrichum* spp. were at a level of 24.936 and 24.320 for soil samples inoculated with a 5000 and 10,000 spore suspension, respectively (Table 4). The Cq values obtained from strawberry fruits inoculated with 100, 1000, 10,000 and 100,000 spores per 1 g ranged from 15.977 to 21.672 for the detection of *Botrytis* spp. after 72- and 0-incubation hours, respectively. The results suggested that the real-time PCR assay could be used for soil and fruits samples assessment in order to detect the important soil-borne fungal pathogens of strawberry.

### 2.6. Validation Assay in Environmental Samples

**Figure 4:**
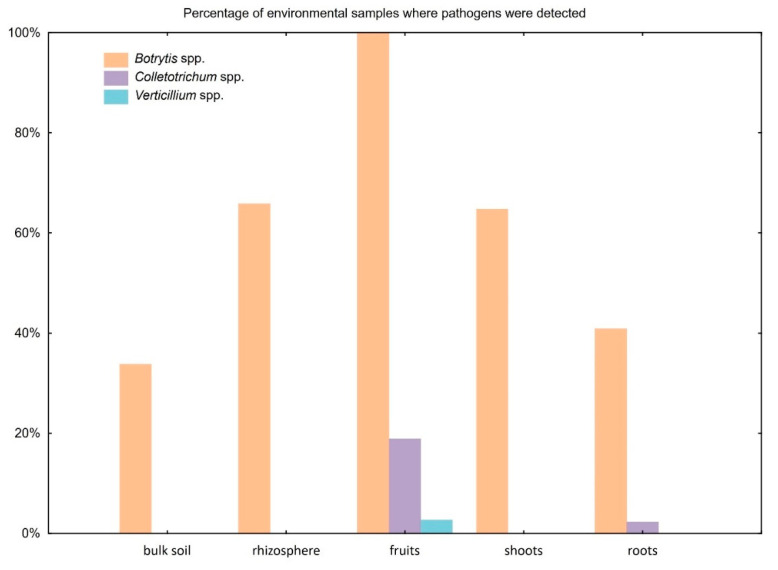
Group column chart representing the percentage of pathogens detected with the developed methodology for each type of environmental samples.

The validation of the method with environmental samples showed that fungi belonging to the *Botrytis* spp. were present within the group of analyzed samples, and were the most abundant in the following groups: bulk soil, roots, shoots, rhizosphere and fruits, with 34%, 41%, 65%, 66% and 100%, respectively (Figure 4). The *Colletotrichum* spp. was detected in 2% of root samples and 19% of fruit samples. Lastly, *Verticillium* spp. was only detected in 3% of fruit samples. 

It is worth to mention that the developed pathogens detection method is suitable for different soil types and plants varieties, as pathogens were detected in acrisol, cambisol, chernozem, fluvisol and regosol and positive detection of pathogens was observed in soil under cultivation of Aprica, Dipred and Honeoye strawberry. However, detection of pathogens in rhizosphere soil included mainly fluvisol soil type for Aprica, Dipred and Honeoye variety, but also cambisol and chernozem for Honeoye and Aprica, respectively. Pathogens were also detected in the rhizosphere of Faith, Marmolada and Rumba strawberries. The pathogens were detected in fruits from organic and conventional plantations, especially in Honeoye variety of strawberry, in shoots of Allegro, Dipred, Honeoye and Rumba, and in roots of Malwina and Marmolada (Table S3). 

## 3. Discussion

Most genera of fungi are ubiquitous and cosmopolitan in soil, and some of them are plant diseases agents. In fungal communities, *Botrytis* spp., *Colletotrichum* spp. and *Verticillium* spp. are found as one of the most significant pathogenic agent for many plants, including strawberry [33]. The identification of plant pathogenic fungi at the genus level by conventional morphological methods requires long-term isolation and incubation for maturation, as it is based on the formation of various structures, such as conidia, sclerotia or ascospores, and also on the differentiation of their microstructure [34]. The rapid detection of phytopathogenic fungi has been the main concern of agriculture for many years, because rapid and efficient methods for the identification of diseases allow for faster and more suitable protective measures in plantations and nurseries. Accordingly, the real-time PCR approach may act as a very useful tool for the detection of phytopathogenic fungi [35]. Thus, the purpose of this study was to develop a method of detection for the three abovementioned phytopathogenic fungal genera in one reaction of real-time PCR.

Thus far, many real-time and multiplex real-time PCR assays for the identification of fungal pathogens in food plantations have been developed. *Verticillium* spp. identification and quantification assays for hop [36] tomato [37], olive [38], strawberry [39] and soil [40] have already been reported. The multiplex reaction has also been researched [35,41,42]. For the *Botrytis* spp., qPCR was used for various plants [25], and a multiplex approach was researched for alfalfa [13]. Lastly, a method for the detection of *Colletotrichum* spp. in strawberry with quantitative PCR has been developed [26,43], as well as duplex [44] and multiplex [45] approaches for olive and soybean, respectively. However, to date, no triplex assay has been developed for three of the phytopathogenic fungi within the same region.

In the presented study, we designed primers and molecular probes and optimized multiplex qPCR reaction for the identification of three important berry pathogens in one run: *Botrytis* spp., *Colletotrichum* spp. and *Verticillium* spp. A new pair of universal fungal primers and three new genus-specific fluorogenic probes were developed for the selected fungi. Multiplex qPCR was optimized and the detection limits for the reaction were also distinguished with DNA isolated from pure strain cultures. The designed assay was specific to all of the chosen fruit pathogens. The detection limit of the reaction was equal to 39 fg/µL for *Botrytis* spp. and *Verticillium* spp. and 156 fg/µL for *Colletotorichum* spp. The research included experiments with mixtures of different strains, but also with samples of soil and fruits artificially contaminated by fungi. We also validated developed detection methods on naturally infested samples of rhizosphere, soil, fruit, roots and shoots of strawberry plants. The results indicate that the method is highly reliable, even in cases of contamination with different types of fungi. Similar results for the PCR method were obtained by Yaguchi et al. (2012) [46] and Pertile et al. (2020) [47] for heat resistant fungi causing food spoilage. Moreover, Feng et al. (2014) [35] indicated that the qPCR assay is useful in the spinach seed industry to detect and quantify the fungal seedborne pathogens of spinach.

In conclusion, a rapid method was developed for detecting *Botrytis*, *Colletotrichum* and *Verticillium* species, which are important diseases-causing organisms in strawberry production.

## 4. Materials and Methods

### 4.1. Phytopathogenic Organisms

Fungal cultures of *Botrytis* spp., *Colletotrichum* spp., *Verticillium* spp. and *Phytophthora* spp., along with other phytopathogenic strains of fungi (Figure 2, Table S1), which were characterized by different morphotypes observed at in vitro cultures, were isolated from organic plantations of strawberries in Poland. In order to isolate the fungal strains, a number of microbiological methods were used, including: (i) direct isolation on different culture media through surface sowing from serial dilutions on the media: potato dextrose agar (PDA), multi-vegetable juice agar (V8), malt extract agar (MEA) and nutrient solution with Bengal rose and antibiotics (Martin medium); (ii) isolation using apple traps, through inoculating green apples of the Granny Smith variety with fragments of infected plant tissues; (iii) isolation consisting of laying strawberry root, shoot and fruit fragments with visible disease symptoms on selective media; (iv) isolation using a trap technique with strawberry leaves, which was based on the arrangement that the root fragments were lined into cuvettes and flooded with water, and the leaves were covered with foil and incubated for 3–5 days. Then, the leaves with necrotic spots were lined on PDA medium. For each method, fungal isolates were finally cultivated on the PDA and incubated at 22 °C for 10 days. Most of the isolated fungi were identified on genus level and some of them on species level (Table S1) based on DNA extraction, with subsequent amplification of the D2 large subunit region of the fungal rDNA [30] and sequencing as described by Frąc et al. (2014) [48] with minor modifications.

### 4.2. Isolation of DNA

#### 4.2.1. DNA Isolation from Pure Culture of Fungi

The DNA used for the identification purposes, as well as specificity of the developed assay derived from the pure strains of the microorganisms, was isolated with the PrepMan Ultra Sample Preparation Reagent (Applied Biosystems by Thermo Fisher Scientific, Waltham, MA, USA). Fragments of mycelia, gathered with the microbiological loops from the pure strains grown on Potato Dextrose Agar (PDA), were diluted in 100 µL of the reagent. Samples were then vortexed for 30 s and heated for 10 min in 100 °C in thermoblock. Next, after 2 min at room temperature, the samples were centrifuged for 2 min in 20,000× *g*. Finally, the samples were diluted 10 times and frozen until used.

For DNA isolation purposes, all fungi chosen for the assay optimization were grown in 15 mL conical flasks with 5 mL of Potato Dextrose Broth (PDB) at 22 °C for 10 days. Then, the probes were centrifuged for 15 min at 4500× *g*, the medium was disposed of and the supernatant was washed with sterile water and centrifuged two times more. The mycelium was then transferred in sterile conditions into 2 mL tubes filled with 0.5 g and 0.25 g glass beads of 3.15 mm and 1.4 mm diameter, respectively. Each tube with mycelium and glass beads also contained 400 µL Lyse F buffer (EURx, Gdańsk, Poland). The samples were then homogenized in the FastPrep-24 instrument (MP Biomedicals) at 4 m/s for 10 s (*Colletotrichum* spp. and *Verticillium* spp.), while for the *Botrytis* spp., homogenization was conducted for 20 s, as those pathogens produced rigid sclerotia in in vitro cultures. Thereafter, genomic DNA isolation with a Plant & Fungi DNA Purification Kit (EURx, Gdańsk, Poland) was performed following the manufacturer’s instruction. Finally, the derived DNA was suspended in 100 µL of Tris-HCl buffer (10 mM Tris-HCl, pH 8.5). The quantity and quality of the obtained genetic material was determined electrophoretically in 2% agarose gel, spectrophotometrically with the NanoDrop 2000 instrument (Thermo Fisher Scientific, Waltham, MA, USA) and fluorometrically with QuantusFluor® DNA dsDNA reagents on a Quantus fluorometer (Promega, Madison, WI, USA). Samples of the DNA were then stored at –22 °C until used.

#### 4.2.2. DNA Isolation from Environmental Samples

DNA extractions from artificially infested soils and fruits were performed using commercially available DNA extraction kits. DNA from the soil samples was extracted with EURx GeneMATRIX Soil DNA Purification KIT (EURx, Gdańsk, Poland), Qiagen DNeasy PowerSoil (Qiagen, Hilden, Germany) and Macherey-Nagel NucleoSpin Soil (MACHEREY-NAGEL, Düren, Germany) kits, using 0.5 g of soil according to the manufacturer’s protocol. Extractions with MP Biomedicals FastDNA Spin Kit for Feces (MP Biomedicals, Solon, OH, USA) were performed according to the protocol of the producer with the modification described below. The same protocol was used for DNA extraction from environmental samples collected from organic plantations of strawberry including roots, shoots, fruits, bulk soil and rhizosphere. Primarily, 0.25 g of plant tissue or 0.5 g of soil were introduced into 2 mL tubes containing a matrix of 1.4 mm ceramic beads, 0.1 mm silica balls and one glass ball 4 mm in diameter. The samples were then washed in sodium phosphate buffer (825 µL) with PLS reagent (275 µL). The samples were centrifuged (5 min, 14,000× *g*) and the supernatant was drained. Sodium phosphate buffer (978 µL) and MT buffer (122 µL) were added, after which the samples were homogenized in a FastPrep24 instrument under the following conditions: 40 s, 6 m/s. The samples were then centrifuged (15 min, 14,000× *g*) and the supernatant was transferred into new tubes containing PPS buffer (250 µL). Then, the samples were mixed by inversion and incubated (10 min at 4 °C). The mixture was centrifuged (2 min, 14,000× *g*) and then the supernatant was transferred to a new 5 mL tube containing Binding Matrix Solution (1 mL). The samples were then mixed on a rotator (5 min), centrifuged (2 min, 14,000× *g*) and the supernatant was drained. The pellet was washed with wash buffer (Wash Buffer 1, 1 mL). The resulting suspension was transferred to a separation column (SPIN Filter) twice, centrifuged (1 min, 14,000× *g*) and the filtrate was discarded. The filter was then washed with a second wash buffer (Wash Buffer 2500 µL), centrifuged (2 min, 14,000× *g*) and the filtrate was discarded. Then, the filter was centrifuged again (2 min, 14,000× *g*), after which the filtrate was transferred into a new tube. Elution buffer (TES, 100 µL) was pipetted onto the filter and centrifuged (2 min, 14,000× *g*). The resulting filtrate, containing extracted DNA, was diluted 10-fold with nuclease-free deionized water. For DNA isolation from strawberry fruits, an EURx GeneMATRIX Soil DNA Purification Kit (EURx, Gdańsk, Poland) and MP Biomedicals FastDNA Spin Kit for Feces (MP Biomedicals, Solon, OH, USA) kits with an input of 0.25 g of strawberry fruit tissue were used.

### 4.3. Oligonucleotide Primers and Probes Design

**Figure 5:**
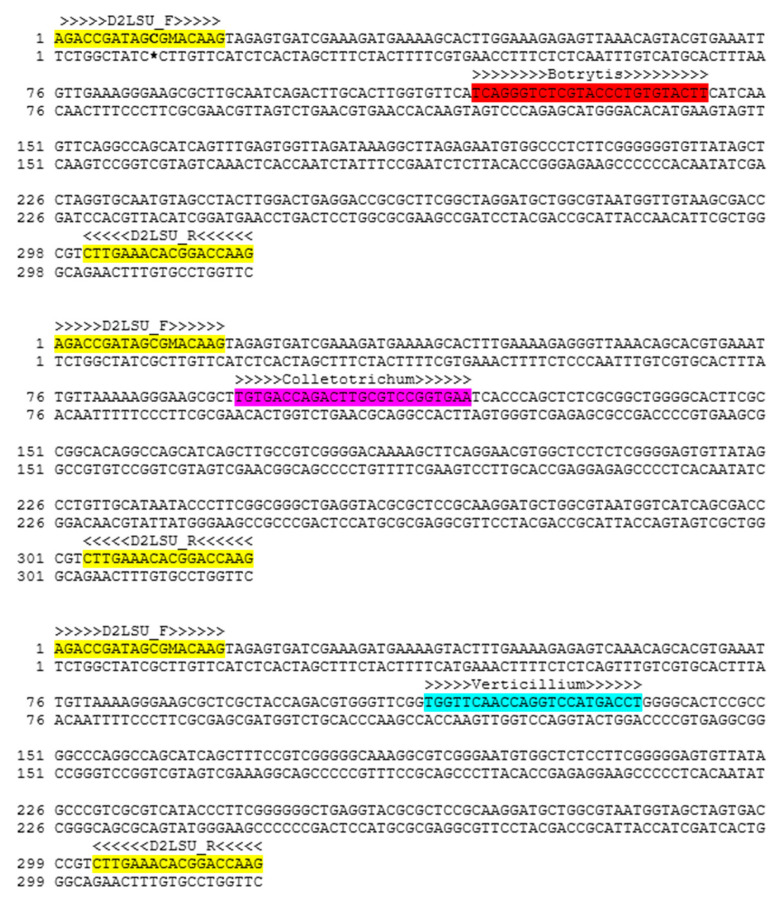
Positioning and orientation of primers (marked as yellow) and fluorogenic probes designed for the real-time PCR within the nucleotide sequence of the D2 region of the large subunit of the rRNA gene for *Botrytis* spp. (marked as red), *Colletotrichum* spp. (marked as pink) and *Verticillium* spp. (marked as blue) (accession #: MT154304.1, MT126802.1 and MT133320.1, respectively).

In order to design primers and probes, 7, 19 and 12 nucleotide sequences of *Botrytis* spp., *Colletotrichum* spp. and *Verticillium* spp., respectively, isolated from an organic plantation of strawberries, were used. The fungi were previously identified through the Sanger sequencing of the D2 region of the large subunit of the rRNA gene (D2 LSU rDNA), and then the sequences were deposited in the NCBI database (Supplementary Table S1). In order to determine the genus-specific and conserved sequences of each pathogen for the primers and probes design, the obtained sequences were aligned with a Multiple Sequence Comparison by Log-Expectation (MUSCLE) algorithm [29,31] and compared with the NCBI GenBank sequence database using the Basic Local Alignment Search Tool (BLAST) [49,50,51,52,53]. After that, Primer3Plus software was used for the oligonucleotide probes design [54]. The experimental design for multiplex quantitative PCR is more complicated than for single reactions. The fluorogenic probes were designed to be highly specific within the genus for the D2 region. The probes used to detect individual targets must contain unique reporter dyes with distinct spectra; therefore, when we had chosen dyes with appropriate excitation wavelengths, we were looking for the dyes which have different emission spectra without their overlapping. Three molecular probes targeting *Botrytis* spp., *Colletotrichum* spp. and *Verticillium* spp. were labeled with 6-carboxyfluorescein (6FAM), carboxytetramethylrhodamine (Tamra) and cyanine (Cy5) fluorescent dyes at the 5’-end, respectively, and Black Hole Quencher-1 (BHQ1), Black Hole Quencher-2 (BHQ2) and BHQ2 as non-fluorescent quenchers at the 3’-end, respectively (Table 1). The specificity of the newly designed probes was further tested in silico using the BLAST algorithm to avoid cross-homology with other microorganisms. Moreover, in order to confirm the specificity of the designed primers, in silico analysis based on sequences from NCBI GenBank (https://www.ncbi.nlm.nih.gov/genbank/) was performed, which included targeted sequences of *Botrytis* spp., *Colletotrichum* spp. and *Verticillium* spp. as well as the other non-targeted fungal sequences of Sclerotiniaceae, Glomerellaceae and Plectosphaerellaceae representatives as outgroups (Table S2). Results were presented as a BLAST Score, E-value and Identities/Query (which corresponds to the number of exact matches in the search and length of the query). The BLAST (alignment) score was calculated by assigning a value of +2 for each aligned pair and –3 for each mismatch and then summing these values. The sequences of the primers and the fluorogenic probes designed for this study are summarized in Table 1. All of the oligonucleotides were synthesized by Genomed S.A. (Warsaw, Poland). Additionally, the positioning of all of the designed fluorogenic probes within the aligned sequences of the chosen fungi are shown in Figure 5.

### 4.4. Development of the Multiplex Real-Time PCR Assay

All of the real-time PCR reactions were carried out in a 7500 Fast thermocycler (Applied Biosystems, Foster City, CA, USA). The reaction mixture consisted of: 10 μL MP qPCR Master Mix (2x) (EURx, Gdańsk, Poland), 0.8 μL (0.4 μM) of each Forward and Reverse primers, 0.2 μL (final concentration 0.1 μM) of fluorogenic probe(s), 25U of thermolabile uracil-N-glycosylase (UNG), 0.25 μL (final concentration 0.31 μM) ROX (EURx, Gdańsk, Poland), 6.35 μL of nuclease-free water (EURx, Gdańsk, Poland) and 2 μL of the template DNA (5 ng/μL); with a total volume of the reaction mix of 20 μL. The real-time PCR conditions were optimized and established as follows: 2 min at 37 °C incubation for UNG activation, one cycle at 95 °C for 12 min for a hot start polymerase activation, then 40 cycles at 95 °C for 5 s and 60 °C for 2 min. All of the reactions were performed in biological triplicates, and a non-template control probe (NTC) was performed with sterilized DirectQ water.

#### 4.4.1. Specificity of the Fluorogenic Probes

In order to determine the specificity of the fluorogenic probes, four selected strains of *Botrytis* sp. (G277/18), *Colletotrichum* sp. (G171/18), *Verticillium* sp. (G296/18) and *Phytophthora* sp. (G408/18)—as a non-specific control pathogen—were selected and tested in a singleplex and then in a multiplex real-time PCR assay. *Phytophthora* species, as a control not-target organism, wereas included into the study, due to the fact that representatives of this pathogen are very often present in soil together with *Verticillium* pathogenic fungi, and sometimes it is difficult to distinguish early disease symptoms caused by these two pathogens. The template DNA of the strains was first diluted to 5 ng/μL. Each reaction mix consisted of the reagents mentioned above, the DNA mixture of all tested fungal pathogens and a separate molecular probe specific for the detection of the desired microorganism with a singleplex PCR approach or the DNA mixture of fungal pathogens and a mixture of fluorogenic probes at an equal amount of 0.2 μL (final concentration 0.1 μM) as the multiplex assay. Additionally, for the assessment of the specificity of the developed probes, the assay was conducted with the use of the DNA isolated from the pure strains of the identified pathogens. In order to evaluate the specificity of each real-time assay, each group of pathogens was tested with each set of primers and probe. The reaction was conducted on the 19 strains if the *Colletotrichum* spp. (G161/18, G162/18, G164/18, G166/18, G167/18, G168/18, G170/18, G171/18, G172/18, G274/18, G350/18, G353/18, G355/18, G356/18, G400/18, G401/18, G404/18, G405/18, G406/18), 7 strains of *Botrytis* spp. (G269/18, G275/18, G276/18, G277/18, G321/18, G322/18, G323/18) and 12 *Verticillium* spp. strains (G293/18, G294/18, G296/18, G297/18, G298/18, G299/18, G302/18, G327/18, G328/18, G330/18, G335/18, G345/18) (Figure 3).

#### 4.4.2. Optimization of the Multiplex Real-Time PCR

For the optimization of the multiplex real-time PCR assay for *Botrytis* spp., *Colletotrichum* spp. and *Verticillium* spp., multiple concentrations of probes (final concentrations 0.1, 0.15, 0.2, 0.25, 0.3, 0.35 μM) and primers (final concentrations 0.1, 0.2, 0.3, 0.4, 0.5, 0.6, 0.7 μM) were tested. The reaction mixture consisted of: 10 μL MP qPCR Master Mix (2x) (EURx, Gdańsk, Poland), which was the tested concentration of primers and fluorogenic probes, 0.25 μL (final concentration 31 μM) ROX (EURx, Gdańsk, Poland) and 4 μL of the pathogen DNA mix (each of the pathogens’ DNA was diluted to 5 ng/μL and combined), and probes were filled with 20 μL of nuclease-free water (EURx, Gdańsk, Poland). The reaction was performed in biological triplicates and the non-template control probe (NTC) was prepared with sterilized DirectQ water.

### 4.5. Detection Limit of Multiplex Reactions

For the determination of the detection limit in the multiplex reaction, a mixture of the three pathogens with an equal DNA concentration of 5 pg/μL was obtained. Then, serial 2-fold dilutions of the mixture were performed and tested, to the lowest concentration of 39 fg/μL. Therefore, in this experiment the following DNA concentrations were tested: 5 pg/µL; 2.5 pg/µL; 1.25 pg/µL; 625 fg/µL; 312 fg/µL; 156 fg/µL; 78 fg/µL and 39 fg/µL. The reaction was performed in biological triplicates and the non-template control probe (NTC) was prepared with sterilized DirectQ water. All designed fluorogenic probes were mixed and used in the same reaction. To show and underline the differences in the detection limit for each tested pathogen, the results are presented as filtered data for *Botrytis* spp., *Colletotrichum* spp. and *Verticillium* spp. (Supplementary Figure S2).

### 4.6. Detection of Fungal Pathogens in Artificially Infested Environmental Samples

The assessment of fungal detection in artificially infested samples was conducted with DNA isolated from the soil and strawberry fruits contaminated with the dilutions of pathogen spores with a specified concentration. The spores were acquired from liquid (*Verticillium* spp.) and solid cultures (*Botrytis* spp. and *Colletotrichum* spp.) and counted in a Thoma counting chamber. Environmental samples including soil and strawberry fruits were inoculated with the appropriate spore suspension. One gram of the soil was contaminated with 1 mL of suspension with a known spore concentration separately for each fungal pathogen: 10,000, 5000, 1000 and 500. One gram of strawberry fruits was artificially infected with 1 mL containing 100,000, 10,000, 1000 and 100 spore suspensions of *Botrytis* spp., as the *Botrytis* spp. is the main concern for strawberry fruits. The pathogen DNA was isolated with the described methods, and the real-time PCR with specific fluorogenic probe and primers was performed to evaluate the detection level of fungal pathogens in artificially contaminated environmental samples. 

### 4.7. Validation Assay in Environmental Samples

The usefulness of the new multiplex real-time PCR assay was evaluated using 244 environmental samples (Supplementary Table S3). Environmental samples used in the real-time PCR assays were provided by farmers who cultivate organic strawberry plantations with different varieties of strawberry plants cultivated on various soil types. Forty-one environmental samples were collected from strawberries rhizosphere and 68 samples were obtained from bulk soil. The origins of the remaining 135 samples were as follows: 37 were collected from strawberries fruits, including 4 samples of fruits from conventional plantations, 44 were roots samples and 54 were shoots of plants samples. Environmental samples were analyzed from naturally infected hosts plants or soil collected in 2018 and 2019 from plantations of strawberries in Poland. Plantations were located at different soil types including fluvisol, chernozem, regosol, acrisol and cambisol with various varieties of strawberry, such as Rumba, Marmolada, Honeoye, Dipred, Aprica, Allegro, Malwina and Faith (Table S3).

### 4.8. Software, Data Collection and Analysis

All real-time PCR fluorescence data was collected and analyzed with 7500 software v 2.0.5 of the 7500 Fast Real-Time PCR System (Foster City, CA, USA). The same software was used to calculate the Cq values. Charts were prepared with STATISTICA v 13.1 (StatSoft Inc., Tulsa, OK, USA) and Microsoft Excel 2013 software (Redmond, WA, USA). Genetic analyses, alignments and phylogenetic analyses were performed with MEGA 7.0.18 software (University Park, PA, USA).

### 4.9. Data Availability

All of the obtained nucleotide sequences are deposited in the GenBank repository (https://www.ncbi.nlm.nih.gov/genbank/) (the accession numbers are grouped together in the Supplementary Materials).

## Figures and Tables

**Table 1:** Sequences of primers and probes, product size of the obtained amplicons for each fungal pathogen and the excitation and fluorescent readings of the probes of the designed oligonucleotides for the detection of selected plant pathogenic fungi in real-time PCR (Patent application P.431988); “N/A” means not applicable.

Primer or Probe Name	Sequence	Product Size (bp)	Excitation/Fluorescence Reading (nm)
D2LSU_F	5′-AGACCGATAGCGmACAAG-3′	N/A	N/A
D2LSU_R	5′-CTTGGTCCGTGTTTCAAG-3′	N/A	N/A
Botr-6FAM	5′-6FAM-TCAGGGTCTCGTACCCTGTGTACTT-BHQ1-3′	322	495/520
Coll-Tamra	5′-Tamra(R)-TGTGACCAGACTTGCGTCCGGTGAA-BHQ2-3′	321	544/576
Vert-Cy5	5′-Cy5-TGGTTCAACCAGGTCCATGACCT-BHQ2-3′	320	643/667

**Table 2:** Mean Cq and SD values of the optimization reactions for the developing concentration of the primers and fluorogenic probes in the real-time PCR (*n* = 3); “–” means lack of amplification.

**Conc. of Fluorogenic Probes**	***Botrytis* spp. (G277/18)**		***Colletotrichum* spp. (G171/18)**		***Verticillium* spp. (G296/18)**	
	**Cq_mean_**	**SD**	**Cq_mean_**	**SD**	**Cq_mean_**	**SD**
0.1 µM	14.343	0.268	18.925	0.482	17.749	0.512
0.15 µM	14.084	0.377	19.286	0.152	18.235	0.417
0.2 µM	12.792	0.895	18.576	0.329	16.942	0.440
0.25 µM	13.930	0.615	18.921	0.733	16.838	0.590
0.3 µM	14.093	0.533	18.814	0.185	20.576	5.697
0.35 µM	13.569	0.455	18.832	0.431	14.898	–
**Conc. of Primers**	**Cq_mean_**	**SD**	**Cq_mean_**	**SD**	**Cq_mean_**	**SD**
0.1 µM	21.421	4.103	–	–	–	–
0.2 µM	15.451	0.095	18.915	0.467	17.459	1.398
0.3 µM	16.478	0.172	18.777	0.142	16.565	0.146
0.4 µM	15.178	0.090	19.067	0.104	17.654	0.487
0.5 µM	15.315	0.321	18.740	0.164	16.872	0.846
0.6 µM	15.389	0.215	18.633	0.040	15.994	0.909
0.7 µM	15.795	0.205	18.821	0.264	19.326	–
0.8 µM	14.963	0.364	18.718	0.233	21.984	10.268

**Table 3:** Mean Cq and SD values for the detection limit of the real-time PCR assay (*n* = 3); “–“ means lack of amplification.

DNA Conc.	*Botrytis* spp. (G277/18)		*Colletotrichum* spp. (G171/18)		*Verticillium* spp. (G296/18)	
	Cq_mean_	SD	Cq_mean_	SD	Cq_mean_	SD
5 pg/µL	28.222	1.659	32.144	1.106	30.659	0.902
2.5 pg/µL	28.076	0.543	31.955	0.155	30.888	0.196
1.25 pg/µL	29.278	0.691	33.200	0.859	31.969	0.817
625 fg/µL	30.668	0.852	34.470	1.659	32.628	0.056
312 fg/µL	31.600	0.950	35.028	1.072	34.037	0.938
156 fg/µL	32.841	–	36.699	0.731	36.391	0.223
78 fg/µL	33.750	2.542	34.012	–	35.219	1.524
39 fg/µL	33.276	0.286	–	–	36.267	0.610

**Table 4:** Cq values of the triplex approach for the detection of artificially infested environmental samples (soil and strawberry); “–” means lack of amplification.

**Time of Incubation**	**Spores per 1 g of Soil**	***Botrytis* spp. (G277/18)**	***Colletotrichum* spp. (G171/18)**	***Verticillium* spp. (G296/18)**
24 h	500	25.513	–	27.732
	1000	24.980	–	26.673
	5000	24.278	24.936	24.306
	10,000	28.166	–	22.491
48 h	500	24.731	–	27.004
	1000	24.642	–	26.356
	5000	24.790	–	24.479
	10,000	25.684	24.320	23.261
**Time of Incubation**	**Spores per 1 g of Strawberry**	***Botrytis* spp. (G277/18)**		
0 h	100	21.672		
	1000	19.289		
	10,000	20.106		
	100,000	19.242		
72 h	100	15.980		
	1000	19.810		
	10,000	21.032		
	100,000	19.597		

## Supplementary Materials

Supplementary materials can be found at https://www.mdpi.com/1422-0067/21/22/8469/s1. Table S1. List of fungal isolates used in this study and obtained from organic plantations of strawberry, with the accession number of the obtained amplicons in the GenBank. Explanations: * LMEM—Laboratory of Molecular and Environmental Microbiology, Institute of Agrophysics, Polish Academy of Sciences (IA PAS) Sample codes in bold are the isolates selected for development of multiplex real-time PCR detection method. Table S2. Specificity of Botr-6FAM Probe (Minimum 3 hits). Results of in silico specificity test of Botr-6FAM probe with megablast searchversus NCBI nt database. Search Included Leotiomycetes fungi. Only organisms with 3 or more hits are shown. Query length of this search was 24, while Maximum BLAST score for this probe is 48.1. The BLAST (alignment) score is calculated by assigning a value of +2 for each aligned pair and -3 for each mismatch and then summing these values. Specificity of Coll-Tamra Probe (Minimum 3 hits). Results of in silico specificity test of Coll-Tamra Probe with megablast search versus NCBI nt database. Search Included Sordariomycetes fungi. Only organisms with 3 or more hits are shown. Query length of this search was 25, while Maximum BLAST score for this probe is 50.1. The BLAST (alignment) score is calculated by assigning a value of +2 for each aligned pair and -3 for each mismatch and then summing these values. Specificity of Vert-Cy5 Probe (Minimum 3 hits). Results of in silico specificity test of Vert-Cy5 Probe with megablast search versus NCBI nt database. Search Included Sordariomycetes fungi. Only organisms with 3 or more hits are shown. Query length of this search was 23, while Maximum BLAST score for this probe is 46.1. The BLAST (alignment) score is calculated by assigning a value of +2 for each aligned pair and -3 for each mismatch and then summing these values. Table S3. Table gathering information on tested environmental samples. Explanations of color marked samples numbers: yellow—detection of *Botrytis* spp., green—detection of Colletotrichum spp., pink—detection of *Botrytis* spp. and *Colletotrichum* spp., red—detection of *Botrytis* spp. and *Verticillium* spp., blue—detection of *Botrytis* spp., *Colletotrichum* spp. and *Verticillium* spp., samples without any color (black)—lack of pathogens detection. Figure S1. Cq values of primer and probe concentration optimisation reactions. Box represents standard error, whiskersrepresent ranges of values (min-max), and the horizontal line in the middle represents mean values (*n* = 3). Figure S2. Detection limit of the phytopathogenic fungi—*Botrytis* spp., *Colletotrichum* spp. and *Verticillium* spp. The reaction was performed as a multiplex approach in three repeats. Each amplification plot is a mean of three repeats. To show and underline differences in the detection limit for each tested pathogen, the results were presented as filtered data for *Botrytis* spp., *Colletotrichum* spp. and *Verticillium* spp.
