# Supplementary material for: Triplex Real-Time PCR Approach for the Detection of Crucial Fungal Berry Pathogens—Botrytis spp., Colletotrichum spp. and Verticillium spp
Source: Int J Mol Sci. 2020 Nov 11;21(22):8469. doi: 10.3390/ijms21228469 (PMC7697166; doi:10.3390/ijms21228469)
Supplement: Supplementary file 1 [file ijms-21-08469-s001.zip › ijms-968610-proofed-supplementary/Supplementary files_final/Figure S1 and S2.docx]

Triplex real-time PCR approach for the detection of crucial fungal berry pathogens - *Botrytis* spp., *Colletotrichum* spp. and *Verticillium* spp.

Dominika G. Malarczyk, Jacek Panek, Magdalena Frąc


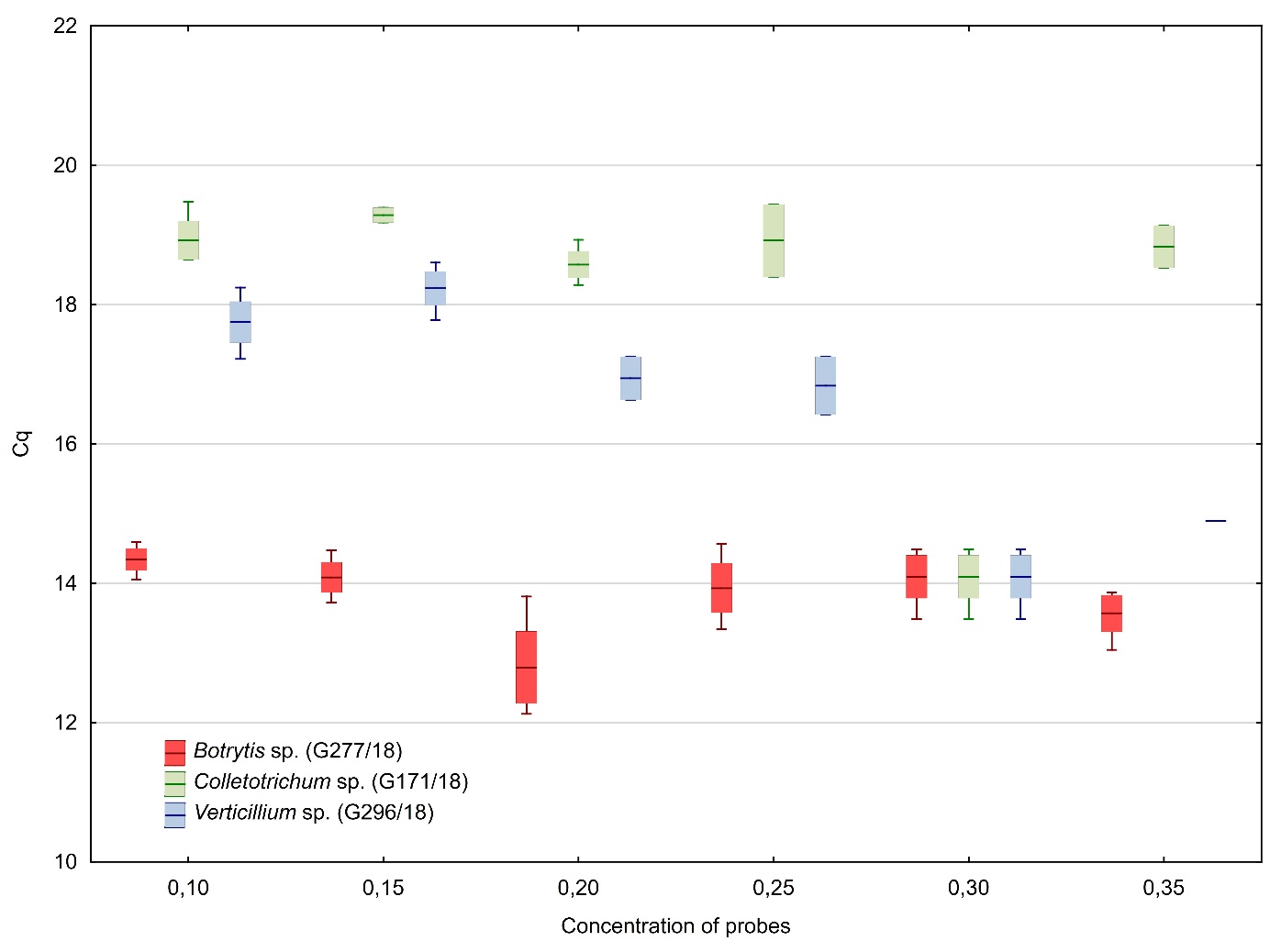


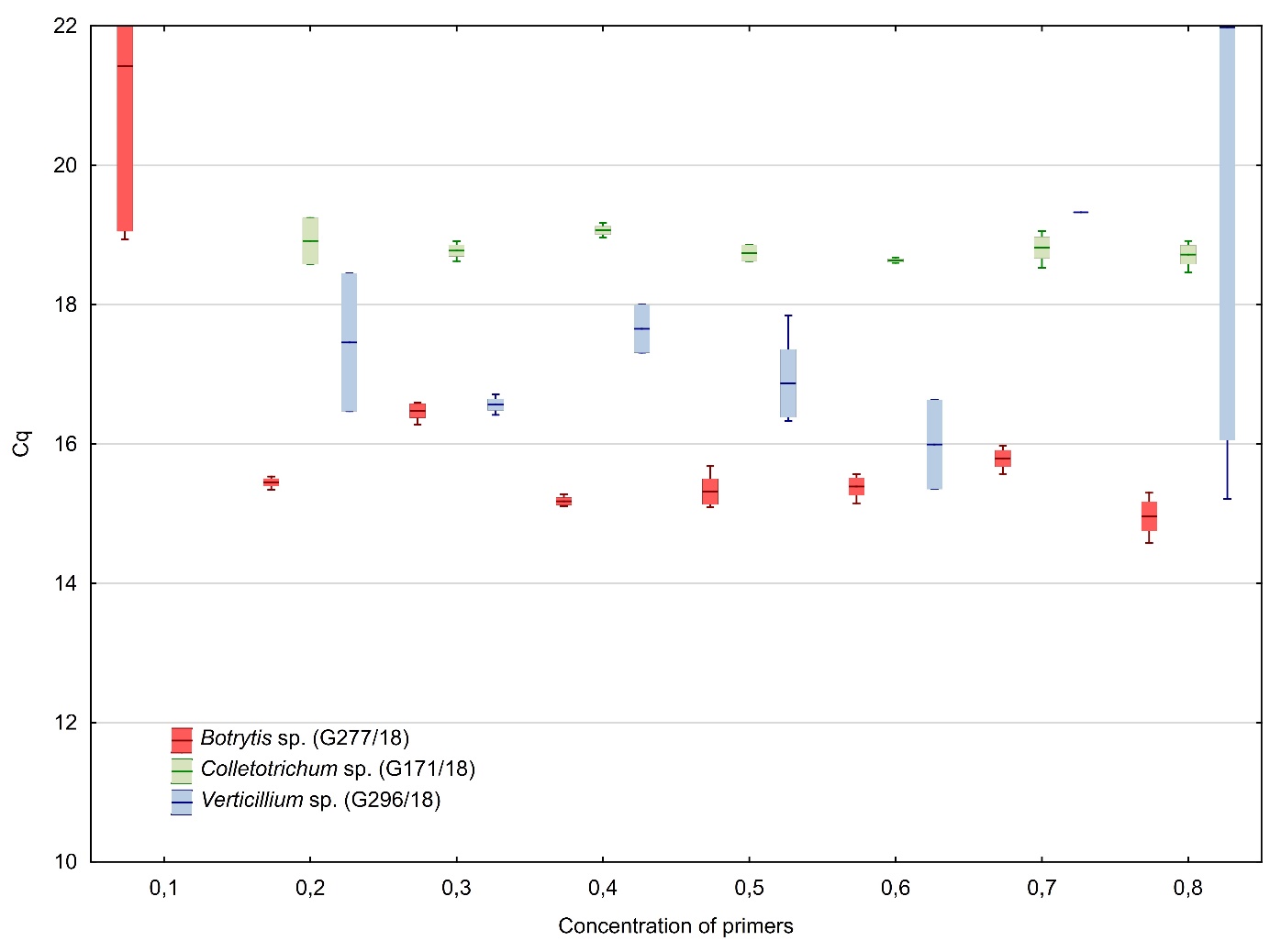


**Figure. S1.** Cq values of primer and probe concentration optimisation reactions. Box represents standard error,, whiskersrepresent ranges of values (min-max), and the horizontal line in the middle represents mean values (*n* = 3).


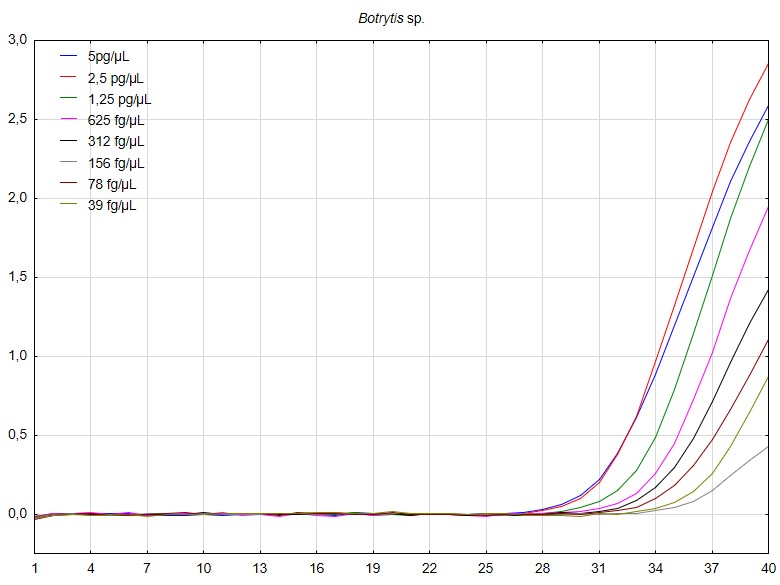


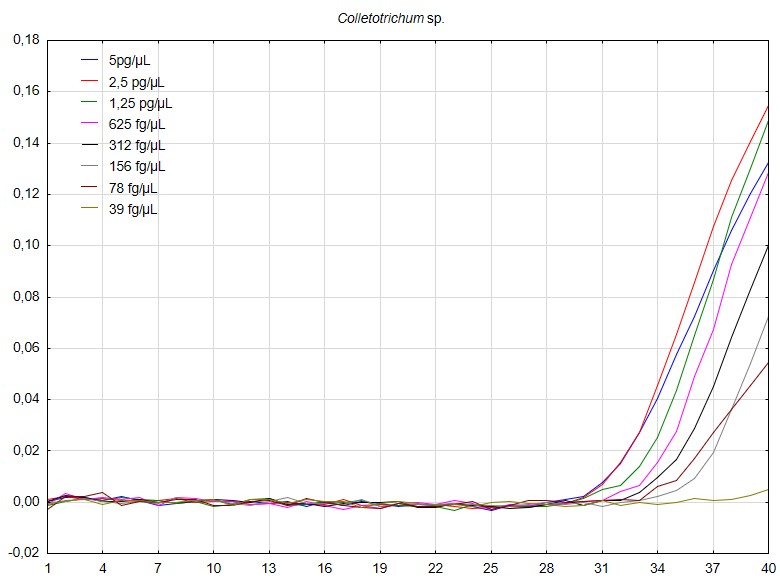


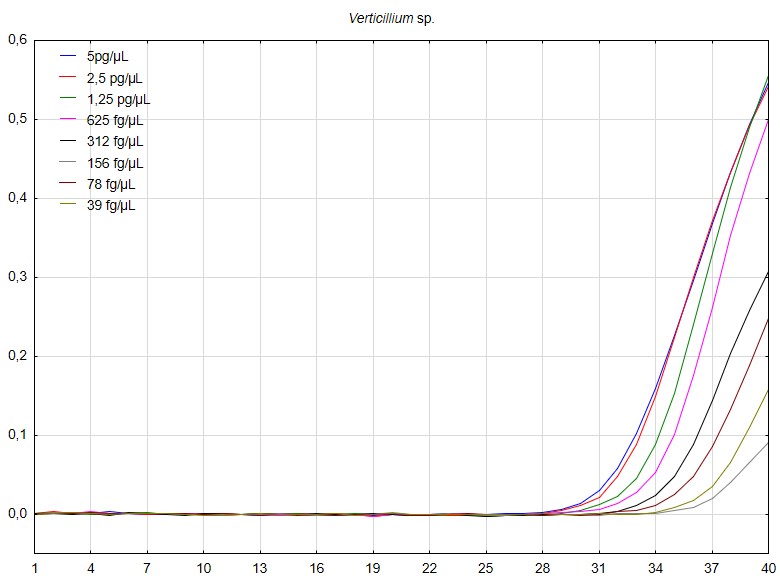


**Figure. S2.** Detection limit of the phytopathogenic fungi – *Botrytis* spp., *Colletotrichum* spp. and *Verticillium* spp. The reaction was performed as a multiplex approach in three repeats. Each amplification plot is a mean of three repeats. To show and underline differences in the detection limit for each tested pathogen, the results were presented as filtered data for *Botrytis* spp., *Colletotrichum* spp. and *Verticillium* spp.
