# Supplementary material for: Triplex Real-Time PCR Approach for the Detection of Crucial Fungal Berry Pathogens—Botrytis spp., Colletotrichum spp. and Verticillium spp
Source: Int J Mol Sci. 2020 Nov 11;21(22):8469. doi: 10.3390/ijms21228469 (PMC7697166; doi:10.3390/ijms21228469)
Supplement: Supplementary file 1 [file ijms-21-08469-s001.zip › ijms-968610-proofed-supplementary/Supplementary files_final/Table S1 and S3.docx]

Triplex real-time PCR approach for the detection of crucial fungal berry pathogens - *Botrytis* spp., *Colletotrichum* spp. and *Verticillium* spp.

Dominika G. Malarczyk, Jacek Panek, Magdalena Frąc

**Supplementary Materials**

**Table S1**. List of fungal isolates used in this study and obtained from organic plantations of strawberry, with the accession number of the obtained amplicons in the GenBank

| Fungal identification | Isolate code LMEM* | Isolation source/institution | Accession number of sequences in GenBank |
| --- | --- | --- | --- |
| *Botrytis* spp. | G269/18 | Strawberry fruits, IA PAS, Poland | MT154301 |
| *Botrytis* spp. | G275/18 | Strawberry roots, IA PAS, Poland | MT154302 |
| *Botrytis* spp. | G276/18 | Strawberry roots, IA PAS, Poland | MT154303 |
| ***Botrytis* spp.** | **G277/18** | Strawberry roots, IA PAS, Poland | MT154304 |
| *Botrytis* spp. | G321/18 | Strawberry roots, IA PAS, Poland | MT154305 |
| *Botrytis* spp. | G322/18 | Strawberry roots, IA PAS, Poland | MT154306 |
| *Botrytis* spp. | G323/18 | Strawberry roots, IA PAS, Poland | MT154307 |
| *Colletotrichum* spp. | G161/18 | Strawberry fruits, IA PAS, Poland | MT126794 |
| *Colletotrichum* spp. | G162/18 | Strawberry fruits, IA PAS, Poland | MT126800 |
| *Colletotrichum* spp. | G164/18 | Strawberry fruits, IA PAS, Poland | MT126799 |
| *Colletotrichum* spp. | G166/18 | Strawberry fruits, IA PAS, Poland | MT126798 |
| *Colletotrichum* spp. | G167/18 | Strawberry fruits, IA PAS, Poland | MT126801 |
| *Colletotrichum* spp. | G168/18 | Strawberry fruits, IA PAS, Poland | MT126804 |
| *Colletotrichum* spp. | G170/18 | Strawberry fruits, IA PAS, Poland | MT126805 |
| ***Colletotrichum* spp.** | **G171/18** | Strawberry fruits, IA PAS, Poland | MT126802 |
| *Colletotrichum* spp. | G172/18 | Strawberry fruits, IA PAS, Poland | MT126803 |
| *Colletotrichum* spp. | G274/18 | Strawberry fruits, IA PAS, Poland | MT126807 |
| *Colletotrichum* spp. | G350/18 | Strawberry shoots, IA PAS, Poland | MT126789 |
| *Colletotrichum* spp. | G353/18 | Strawberry shoots, IA PAS, Poland | MT126796 |
| *Colletotrichum* spp. | G355/18 | Strawberry shoots, IA PAS, Poland | MT126791 |
| *Colletotrichum* spp. | G356/18 | Strawberry shoots, IA PAS, Poland | MT126792 |
| *Colletotrichum* spp. | G400/18 | Strawberry shoots, IA PAS, Poland | MT126790 |
| *Colletotrichum* spp. | G401/18 | Strawberry shoots, IA PAS, Poland | MT126793 |
| *Colletotrichum* spp. | G404/18 | Strawberry shoots, IA PAS, Poland | MT126797 |
| *Colletotrichum* spp. | G405/18 | Strawberry shoots, IA PAS, Poland | MT126806 |
| *Colletotrichum* spp. | G406/18 | Strawberry shoots, IA PAS, Poland | MT126795 |
| *Verticillium* spp. | G293/18 | Strawberry roots, IA PAS, Poland | MT133324 |
| *Verticillium* spp. | G294/18 | Strawberry roots, IA PAS, Poland | MT133317 |
| ***Verticillium* spp.** | **G296/18** | Strawberry roots, IA PAS, Poland | MT133320 |
| *Verticillium* spp. | G297/18 | Strawberry roots, IA PAS, Poland | MT133316 |
| *Verticillium* spp. | G298/18 | Strawberry roots, IA PAS, Poland | MT133318 |
| *Verticillium* spp. | G299/18 | Strawberry roots, IA PAS, Poland | MT133319 |
| *Verticillium* spp. | G302/18 | Strawberry roots, IA PAS, Poland | MT133314 |
| *Verticillium* spp. | G327/18 | Strawberry roots, IA PAS, Poland | MT133327 |
| *Verticillium* spp. | G328/18 | Strawberry roots, IA PAS, Poland | MT133326 |
| *Verticillium* spp. | G330/18 | Strawberry roots, IA PAS, Poland | MT133315 |
| *Verticillium* spp. | G335/18 | Strawberry roots, IA PAS, Poland | MT133322 |
| *Verticillium* spp. | G345/18 | Strawberry roots, IA PAS, Poland | MT133323 |
| ***Phytophthora* spp.** | **G408/18** | Strawberry roots, IA PAS, Poland | MT126670 |
| *Phytophthora* spp. | G368/18 | Research Institute of Horticulture | MT558571 |
| *Phytophthora cactorum* | G369/18 | Research Institute of Horticulture | MT558729 |
| *Phytophthora cinamomi* | G372/18 | Research Institute of Horticulture | - |
| *Phytophthora* spp. | G373/18 | Research Institute of Horticulture | - |
| *Phytophthora cactorum* | G408/18 | Strawberry roots, IA PAS, Poland | MT126670.1 |
| *Phytophthora pseudotsugae* | G409/18 | Strawberry roots, IA PAS, Poland | MT126671.1 |
| *Phytophthora* spp. | G412/18 | Strawberry rhizosphere, IA PAS, Poland | MT126672.1 |
| *Phytophthora* spp. | G413/18 | Strawberry rhizosphere, IA PAS, Poland | MT126673.1 |
| *Phytophthora* spp. | G415/18 | Strawberry rhizosphere, IA PAS, Poland | MT126674.1 |
| *Phytophthora* spp. | G416/18 | Strawberry rhizosphere, IA PAS, Poland | MT126675.1 |
| *Phytophthora* spp. | G417/18 | Strawberry rhizosphere, IA PAS, Poland | MT126676.1 |
| *Phytophthora* spp. | G418/18 | Strawberry rhizosphere, IA PAS, Poland | MT126677.1 |
| *Phytophthora* spp. | G419/18 | Strawberry roots, IA PAS, Poland | MT126678.1 |
| *Phytophthora* spp. | G420/18 | Strawberry roots, IA PAS, Poland | MT126679.1 |
| *Phytophthora* spp. | G421/18 | Strawberry roots, IA PAS, Poland | MT126680.1 |
| *Phytophthora* spp. | G429/18 | Strawberry roots, IA PAS, Poland | MT126681.1 |
| *Phytophthora* spp. | G430/18 | Strawberry roots, IA PAS, Poland | MT126682.1 |
| *Phytophthora* spp. | G431/18 | Strawberry roots, IA PAS, Poland | MT126683.1 |
| *Phytophthora* spp. | G432/18 | Strawberry roots, IA PAS, Poland | MT126684.1 |
| *Phytophthora* spp. | G434/18 | Strawberry roots, IA PAS, Poland | MT126685.1 |
| *Phytophthora* spp. | G437/18 | Strawberry roots, IA PAS, Poland | MT126686.1 |
| *Phytophthora* spp. | G439/18 | Strawberry roots, IA PAS, Poland | MT126687.1 |
| *Phytophthora* spp. | G440/18 | Strawberry roots, IA PAS, Poland | MT126688.1 |
| *Phytophthora* spp. | G441/18 | Strawberry roots, IA PAS, Poland | MT126689.1 |
| *Phytophthora* spp. | G442/18 | Strawberry roots, IA PAS, Poland | MT126690.1 |
| *Allantophoma* spp. | G320/18 | Strawberry roots, IA PAS, Poland | MW175259 |
| *Alternaria* spp. | G211B/18 | Strawberry leaves, IA PAS, Poland | MT154530.1 |
| *Alternaria* spp. | G216/18 | Strawberry leaves, IA PAS, Poland | MT154531.1 |
| *Alternaria* spp. | G242/18 | Strawberry leaves, IA PAS, Poland | MT154532.1 |
| *Alternaria* spp. | G262/18 | Strawberry shoots, IA PAS, Poland | MT154533.1 |
| *Alternaria* spp. | G305/18 | Strawberry roots, IA PAS, Poland | MW175255 |
| *Alternaria* spp. | G306/18 | Strawberry roots, IA PAS, Poland | MT154534.1 |
| *Alternaria* spp. | G307/18 | Strawberry roots, IA PAS, Poland | MT154535.1 |
| *Alternaria* spp. | G308/18 | Strawberry roots, IA PAS, Poland | MT154536.1 |
| *Alternaria* spp. | G309/18 | Strawberry roots, IA PAS, Poland | MT154537.1 |
| *Alternaria* spp. | G310/18 | Strawberry roots, IA PAS, Poland | MT154538.1 |
| *Alternaria* spp. | G313/18 | Strawberry roots, IA PAS, Poland | MT154539.1 |
| *Alternaria* spp. | G314/18 | Strawberry roots, IA PAS, Poland | MT154540.1 |
| *Alternaria* spp. | G357/18 | Strawberry shoots, IA PAS, Poland | MT154541.1 |
| *Alternaria* spp. | G433/18 | Strawberry shoots, IA PAS, Poland | MT154542.1 |
| *Cadophora* spp. | G325/18 | Strawberry roots, IA PAS, Poland | MW175260 |
| *Cadophora* spp. | G333/18 | Strawberry roots, IA PAS, Poland | MW175262 |
| *Cladosporium* spp. | G300/18 | Strawberry roots, IA PAS, Poland | MW175251 |
| *Cladosporium* spp. | G339/18 | Strawberry roots, IA PAS, Poland | MW175266 |
| *Cladosporium* spp. | G445/18 | Strawberry shoots, IA PAS, Poland | MW175284 |
| *Clonostachys* spp. | G443/18 | Strawberry shoots, IA PAS, Poland | MW175282 |
| *Coniella solicola* | G446/18 | Strawberry shoots, IA PAS, Poland | MW175285 |
| *Coniothyrium* spp. | G278/18 | Strawberry roots, IA PAS, Poland | MW175237 |
| *Fusarium* spp. | G260/18 | Strawberry shoots, IA PAS, Poland | MW175234 |
| *Fusarium* spp. | G261/18 | Strawberry shoots, IA PAS, Poland | MW175235 |
| *Fusarium* spp. | G263/18 | Strawberry shoots, IA PAS, Poland | MW175236 |
| *Fusarium* spp. | G290/18 | Strawberry roots, IA PAS, Poland | MW175247 |
| *Fusarium* spp. | G291/18 | Strawberry roots, IA PAS, Poland | MW175248 |
| *Fusarium* spp. | G304/18 | Strawberry roots, IA PAS, Poland | MW175254 |
| *Fusarium* spp. | G311/18 | Strawberry roots, IA PAS, Poland | MW175256 |
| *Fusarium* spp. | G312/18 | Strawberry roots, IA PAS, Poland | MW175257 |
| *Fusarium* spp. | G358/18 | Strawberry shoots, IA PAS, Poland | - |
| *Fusarium* spp. | G403/18 | Strawberry shoots, IA PAS, Poland | MW175271 |
| *Fusarium oxysporum* | G435/18 | Strawberry shoots, IA PAS, Poland | MW175280 |
| *Fusarium oxysporum* | G438/18 | Strawberry shoots, IA PAS, Poland | MW175281 |
| *Fusarium oxysporum* | G447/18 | Strawberry shoots, IA PAS, Poland | MW175286 |
| *Gnomonia radicicola* | G444/18 | Strawberry shoots, IA PAS, Poland | MW175283 |
| *Gnomoniopsis* spp. | G279/18 | Strawberry roots, IA PAS, Poland | MW175238 |
| *Gnomoniopsis* spp. | G280/18 | Strawberry roots, IA PAS, Poland | MW175239 |
| *Gnomoniopsis* spp. | G422/18 | Strawberry shoots, IA PAS, Poland | MW175275 |
| *Gnomoniopsis* spp. | G423/18 | Strawberry shoots, IA PAS, Poland | MW175276 |
| *Ilyonectria* spp. | G337/18 | Strawberry roots, IA PAS, Poland | MW175264 |
| *Metschnikowia* spp. | G402/18 | Strawberry shoots, IA PAS, Poland | MW175270 |
| *Mortierella* spp. | G181/18 | Strawberry leaves, IA PAS, Poland | MW175232 |
| *Mucor* spp. | G264/18 | Strawberry shoots, IA PAS, Poland | - |
| *Mucor* spp. | G265/18 | Strawberry shoots, IA PAS, Poland | - |
| *Mucor* spp. | G266/18 | Strawberry shoots, IA PAS, Poland | - |
| *Mucor* spp. | G267/18 | Strawberry shoots, IA PAS, Poland | - |
| *Mucor* spp. | G270/18 | Strawberry shoots, IA PAS, Poland | - |
| *Mucor* spp. | G271/18 | Strawberry shoots, IA PAS, Poland | - |
| *Mucor* spp. | G272/18 | Strawberry shoots, IA PAS, Poland | - |
| *Mucor* spp. | G273/18 | Strawberry shoots, IA PAS, Poland | - |
| *Mycosphaerella* spp. | G284/18 | Strawberry roots, IA PAS, Poland | MW175243 |
| *Mycosphaerella* spp. | G285/18 | Strawberry roots, IA PAS, Poland | MW175244 |
| *Mycosphaerella* spp. | G287/18 | Strawberry roots, IA PAS, Poland | MW175245 |
| *Penicillium* spp. | G259/18 | Strawberry shoots, IA PAS, Poland | MK801768.1 |
| *Penicillium* spp. | G414/18 | Strawberry shoots, IA PAS, Poland | MW175274 |
| *Pestalotiopsis* spp. | G346/18 | Strawberry roots, Research Institute of Horticulture | MW175267 |
| *Pestalotiopsis* spp. | G410/18 | Strawberry roots, IA PAS, Poland | MW175272 |
| *Pestalotiopsis* spp. | G411/18 | Strawberry roots, IA PAS, Poland | MW175273 |
| *Pestalotiopsis* spp. | G424/18 | Strawberry roots, IA PAS, Poland | MW175277 |
| *Pestalotiopsis* spp. | G425/18 | Strawberry roots, IA PAS, Poland | MW175278 |
| *Phanerochaete* spp. | G295/18 | Strawberry roots, IA PAS, Poland | MW175250 |
| *Phialophora* spp. | G281/18 | Strawberry roots, IA PAS, Poland | MW175240 |
| *Phialophora* spp. | G282/18 | Strawberry roots, IA PAS, Poland | MW175241 |
| *Phialophora* spp. | G301/18 | Strawberry roots, IA PAS, Poland | MW175252 |
| *Phialophora* spp. | G317/18 | Strawberry roots, IA PAS, Poland | MW175258 |
| *Phialophora* spp. | G351/18 | Strawberry shoots, IA PAS, Poland | MW175268 |
| *Phialophora* spp. | G352/18 | Strawberry shoots, IA PAS, Poland | MW175269 |
| *Phlebia* spp. | G283/18 | Strawberry roots, IA PAS, Poland | MW175242 |
| *Phlebia* spp. | G334/18 | Strawberry roots, IA PAS, Poland | MW175263 |
| *Phoma* spp. | G250/18 | Strawberry leaves, IA PAS, Poland | MW175233 |
| *Phoma* spp. | G303/18 | Strawberry roots, IA PAS, Poland | MW175253 |
| *Phoma* spp. | G331/18 | Strawberry roots, IA PAS, Poland | MW175261 |
| *Pilidium* spp. | G191/18 | Strawberry fruit, IA PAS, Poland | MT555756.1 |
| *Pilidium* spp. | G193/18 | Strawberry fruit, IA PAS, Poland | MT555757.1 |
| *Pilidium* spp. | G197/18 | Strawberry fruit, IA PAS, Poland | - |
| *Pilidium* spp. | G199/18 | Strawberry fruit, IA PAS, Poland | MT555760.1 |
| *Pilidium* spp. | G200/18 | Strawberry fruit, IA PAS, Poland | MT555761.1 |
| *Pilidium* spp. | G201/18 | Strawberry fruit, IA PAS, Poland | MT555762.1 |
| *Pilidium* spp. | G204/18 | Strawberry fruit, IA PAS, Poland | MT555763.1 |
| *Pilidium* spp. | G205/18 | Strawberry fruit, IA PAS, Poland | MT555764.1 |
| *Pilidium* spp. | G206/18 | Strawberry fruit, IA PAS, Poland | MT555765.1 |
| *Pilidium* spp. | G207/18 | Strawberry fruit, IA PAS, Poland | MT555766.1 |
| *Pseudeurotium* spp. | G292/18 | Strawberry roots, IA PAS, Poland | MW175249 |
| *Pythium spiculum* | G426/18 | Strawberry rhizosphere, IA PAS, Poland | MW175279 |
| *Sarocladium* spp. | G338/18 | Strawberry roots, IA PAS, Poland | MW175265 |
| *Talaromyces* spp. | G289/18 | Strawberry roots, IA PAS, Poland | MW175246 |

* LMEM – Laboratory of Molecular and Environmental Microbiology, Institute of Agrophysics, Polish Academy of Sciences (IA PAS)
Sample codes in bold are the isolates selected for development of multiplex real-time PCR detection method

**Table 2.** Table gathering information on tested environmental samples. Explnations of colour marked samples numbers: yellow – detection of *Botrytis* spp., green – detection of *Colletotrichum* spp., pink – detection of *Botrytis* spp. and *Colletotrichum* spp., red – detection of *Botrytis* spp. and *Verticillium* spp., blue – detection of *Botrytis* spp., *Colletotrichum* spp. and *Verticillium* spp., samples without any colour (black) – lack of pathogens detection.

| Type of samples | Variety of strawberry | Soil type | Codes of sample |
| --- | --- | --- | --- |
| bulk soil | - | - | 334/19, 335/19 |
| bulk soil | - | - | 702/19 |
| bulk soil | - | - | 726/19 |
| bulk soil | - | - | **727/19** |
| bulk soil | Aprica | fluvisol | **348/19, 350/19** |
| bulk soil | Aprica | chernozem | 538/19A, 538/19B, 538/19C |
| bulk soil | Aprica | chernozem | **380/19, 382/19, 384/19** |
| bulk soil | Aprica | regosol | 55/19A, 55/19B, 55/19C |
| bulk soil | Aprica | regosol | **32/19A, 32/19B, 32/19C** |
| bulk soil | Aprica | - | 452/19, 454/19, 456/19 |
| bulk soil | Aprica | acrisol | **45/19A, 45/19B, 45/19C** |
| bulk soil | Aprica & Honeoye | fluvisol | 48/19A, 48/19**,** 48/19C |
| bulk soil | Dipred | fluvisol | 53/19A, 53/19B, 53/19C |
| bulk soil | Dipred | fluvisol | **358/19, 360/19** |
| bulk soil | Dipred | - | 440/19, **442/19**, 444/19 |
| bulk soil | Dipred | acrisol | 57/19A, 57/19B, 57/19C |
| bulk soil | Honeoye | cambisol | **368/19, 370/19, 372/19** |
| bulk soil | Honeoye | fluvisol | **340/19, 341/19** |
| bulk soil | Honeoye | acrisol | 43/19A, 43/19B, 43/19C |
| bulk soil | Honeoye | acrisol | **539/19A, 539/19B, 539/19C** |
| bulk soil | Honoeye | - | 464/19, 466/19, 468/19 |
| bulk soil | Honoeye | - | **392/19, 396/19** |
| bulk soil | Honoeye | - | 416/19, 418/19, 420/19 |
| bulk soil | Honoeye | - | 403/19, **405/19**, 407/19 |
| bulk soil | Honoeye | - | **428/19, 430/19**, 432/19 |
| bulk soil | Malwina | - | 703/19 |
| fruit | Honeoye | - | **514/19A, 514/19B, 514/19C** |
| fruit | Honeoye | - | **522/19A, 522/19B, 522/19C** |
| fruit | Honeoye | - | **530/19A, 530/19B, 530/19C, 531/19A, 531/19B, 531/19C,** **532/19A,** **532/19B,** **532/19C, 533/19A,** **533/19B, 533/19C, 534/19A, 534/19B, 534/19C, 535/19A, 535/19B, 535/19C,** **536/19A, 536/19B, 536/19C, 537/19A, 537/19B, 537/19C** |
| fruit | organic | - | **246/19** |
| fruit | Honeoye | - | **244/19,** **245/19** |
| fruit | conventional | - | **241/19**; **242/19, 243/19,** **248/19** |
| rhizosphere | Honoeye | - | **404/19, 406/19, 408/19** |
| rhizosphere | Aprica | chernozem | **379/19, 381/19, 383/19** |
| rhizosphere | Honeoye | cambisol | **367/19, 369/19, 371/19** |
| rhizosphere | Allegro | - | 718/19 |
| rhizosphere | Aprica | fluvisol | **347/19**; 349/19; 351/19 |
| rhizosphere | Aprica | - | 451/19, **453/19,** 455/19 |
| rhizosphere | Dipred | fluvisol | **357/19**, 359/19, **361/19** |
| rhizosphere | Dipred | - | 439/19, 441/19, **443/19** |
| rhizosphere | Faith | - | **706/19** |
| rhizosphere | Faith | - | **707/19** |
| rhizosphere | Honeoye | - | **704/19** |
| rhizosphere | Honeoye | fluvisol | 337/19, **338/19, 339/19** |
| rhizosphere | Honeoye | - | **391/19, 393/19, 395/19** |
| rhizosphere | Honoeye | - | 463/19, 465/19, 467/19 |
| rhizosphere | Honoeye | - | 415/19, **417/19, 419/19** |
| rhizosphere | Honoeye | - | **427/19, 429/19, 431/19** |
| rhizosphere | Marmolada | - | **723/19, 724/19** |
| rhizosphere | Rumba | - | 708/19 |
| rhizosphere | Rumba | - | **716/19** |
| roots | Aprica | - | 352/19K, 354/19K, 356/19K |
| roots | Aprica | - | 457/19K, 459/19K, 461/19K |
| roots | Aprica | - | 481/19K, 483/19K, 485/19K |
| roots | Aprica | - | 493/19K, 495/19K, 497/19K |
| roots | Aprica | - | 385/19K, 387/19K, 389/19K |
| roots | Dipred | - | 445/19K, 447/19K, 449/19K |
| roots | Dipred | - | 362/19K, 364/19K, 366/19K, |
| roots | Honeoye | - | 469/19K, 471/19K, 473/19K |
| roots | Honeoye | - | 342/19K, 343/19K, 344/19K, |
| roots | Honeoye | - | 397/19K, 399/19K, 401/19K |
| roots | Honeoye | - | 421/19K, 423/19K, 425/19K |
| roots | Honeoye | - | 433/19K, 435/19K, 437/19K |
| roots | Honeoye | - | 410/19K, 412/19K, 414/19K |
| roots | Honeoye | - | 373/19K, 375/19K, 377/19K |
| roots | Malwina | - | **705/19** |
| roots | Marmolada | - | **722/19** |
| shoots | - | - | 333/19, 336/19 |
| shoots | Allegro | - | **714/19** |
| shoots | Allegro | - | 717/19 |
| shoots | Aprica | - | 352/19L, 354/19L, 356/19L |
| shoots | Aprica | - | 457/19L, 459/19L, 461/19L |
| shoots | Aprica | - | 481/19L, 483/19L, 485/19L |
| shoots | Aprica | - | 493/19L, 495/19L, 497/19L |
| shoots | Aprica | - | 385/19L, 387/19L, 389/19L |
| shoots | Dipred | - | **725/19** |
| shoots | Dipred | - | 445/19L, 447/19L, 449/19L |
| shoots | Dipred | - | 362/19L, 364/19L, 366/19L |
| shoots | Honeoye | - | **712/19** |
| shoots | Honeoye | - | **247/19** |
| shoots | Honeoye | - | 469/19L, 471/19L, 473/19L |
| shoots | Honeoye | - | 342/19L, 343/19L, 344/19L, |
| shoots | Honeoye | - | 397/19L, 399/19L, 401/19L |
| shoots | Honeoye | - | 421/19L, 423/19L, 425/19L |
| shoots | Honeoye | - | 433/19L, 435/19L, 437/19L |
| shoots | Honeoye | - | 410/19L, 412/19L, 414/19L |
| shoots | Honeoye | - | 373/19L, 375/19L, 377/19L |
| shoots | Marmolada | - | 720/19, 721/19 |
| shoots | Rumba | - | **713/19** |
| shoots | Rumba | - | **715/19** |
